# Scrolling to wisdom: The impact of social media news exposure on knowledge perception

**DOI:** 10.3758/s13423-025-02786-3

**Published:** 2025-12-10

**Authors:** Federica Ruzzante, Gustavo Cevolani, Folco Panizza

**Affiliations:** MoMiLab Research Unit, IMT School for Advanced Studies Lucca, Lucca, Italy

**Keywords:** Illusion of knowledge, Social media, Metacognition, News exposure

## Abstract

The present study explores the effect of exposure to news in a social media environment on people’s perceived knowledge and on the “Illusion of knowledge” —that is, the overestimation of perceived knowledge relative to actual knowledge. Using a mixed, within-subjects design, participants (*N* = 828) engaged in a two-session study featuring pre- and post-exposure assessments. Participants scrolled through a social media newsfeed and completed assessments of perceived and actual knowledge. We hypothesized that (1) social media exposure would increase perceived knowledge, (2) this increase would not align with actual knowledge, and (3) higher topic involvement would exacerbate these effects. While most of the preregistered hypotheses did not receive clear statistical support, some effects were consistent with our predictions. Perceived knowledge increased over time, suggesting a general exposure effect; however, differences between exposed and non-exposed topics were not statistically significant, possibly due to test effects or limited engagement with the platform. A strong illusion of knowledge was observed across topics, suggesting robust overestimation. This high baseline may have created a ceiling that constrained the detection of exposure effects. This study was conducted as a Registered Report (https://doi.org/10.24072/pci.rr.100986), accepted in principle prior to data collection. To our knowledge, it represents one of the most ecologically valid attempts in the literature to simulate social media exposure in an experimental setting.

**Supplementary Information:**

The online version contains supplementary material available at 10.3758/s13423-025-02786-3.

## Introduction

The research in metacognition over the past decades has highlighted a distinction between what is knowledge and what is the mere feeling of knowledge (Koriat & Lieblich, 1974). The familiarity with an object or a topic, and the accessibility that follows, is often used as a heuristic to evaluate our own knowledge (Koriat, 2000). Usually, this metacognitive process results in an overestimation of one’s perceived knowledge, leading to a false sense of understanding known in the literature as the illusion of knowledge (IoK; Glenberg et al., 1982).

Such psychological effect has been first operationalized by Glenberg and colleagues (1982). In an empirical study about text understanding, they observed that many of the participants overrated their comprehension whilst failing to detect the contradictions in the text, even when explicitly instructed to do so. In another series of experiments, Rozenblit and Keil (2002) asked participants to rate their knowledge of several topics used as stimuli at different times. The comparison of the ratings between time stops revealed that participants’ perceived knowledge had significantly decreased after they were instructed to provide a detailed explanation of the topic and after being asked to answer a closed-ended question about it. The result was particularly strong for familiar topics, like devices and objects commonly used by participants. The differences in the ratings were explained in terms of availability: when faced with a cue, like a label for a topic or a phenomenon, people tend to build a mental representation of it. This mental image can thus be easily accessed through a perception-like rather than an inferential process (Rozenblit & Keil, 2002); the ease with which people access information, either due to familiarity or ease of interpretation, is thought to trigger heuristic processes and biases (Kahneman & Tversky, 1982). However, when asked to provide a detailed explanation, individuals must engage in inference and reasoning to process the relevant information. This interpretation has also been corroborated by the evidence that participants with a more analytical reasoning style are less susceptible to the IoK: the higher their score in the cognitive reflection task (Frederick, 2005), the more accurate their assessment of perceived knowledge (Fernbach, Sloman, et al., 2013). Low scores in Need for Cognition (Cacioppo & Petty, 1982) were also found to be correlated with greater and unjustified overconfidence (Weber & Koehler, 2017).

The IoK has been consistently found in several domains, concerning, for instance, scientific topics (Rozenblit & Keil, 2002), policy understanding (Rabb et al., 2021), political competence (Leonhard et al., 2020; Weber & Koehler, 2017), mental disorders (Zeveney & Marsh, 2016), action performance (Kardas & O’Brien, 2018), genetically modified foods (Fernbach et al., 2019), consumer preferences (Fernbach, Sloman, et al., 2013), and also COVID-19 (Granderath et al., 2021).

IoK becomes particularly relevant in the context of political discourses about the ability of news media to influence public opinion and convey knowledge. The relationship between news exposure and perceived versus actual knowledge was at the centre of a correlational study about a gubernatorial election campaign in Michigan (Park, 2001). As well as confirming the discrepancy between factual and perceived knowledge, the author found a correlation between news consumption and the IoK, suggesting that news consumption per se does not increase political knowledge, but it is likely to increase the misperception of being well-informed. A further interesting result from this analysis is that participants who felt more involved in the issues covered by the media had a stronger illusion.

Recently, research has focused on testing whether the effect of traditional news exposure on the IoK also translates into social media platforms. It is reasonable to expect that in such environments, where attention is constantly challenged by a large amount of information, people are cognitively impoverished (Simon, 1971) and, therefore, more likely to reason via mental shortcuts. Social media represent a unique environment for users’ reasoning and judgment (Lorenz-Spreen et al., 2020): the overabundance of information on the web is an amount of data impossible to handle for human attention, challenging the quality of users’ decisions (Hills, 2019).

Social media may increase the susceptibility to cognitive biases in general and to the illusions of knowledge in particular. Indeed, there is robust evidence of an inconsistent relationship between online news exposure and increased political knowledge. Gil de Zúñiga et al. (2017) hypothesized that many individuals might have a perception of being well-informed by the mere passive exposure to the news shared by others on social media. This “news-finds-me” perception might prevent people from actively seeking for news from other sources of information (e.g., traditional media). The results of their study confirmed that participants who had the perception of being well-informed were actually less knowledgeable than those who did not hold such belief. This finding has been further validated by survey data that examined the correlation between social media use and political knowledge and whose results suggest that social media use hinders, rather than enhances, users’ learning while at the same time fostering a misperception of knowledge (Cacciatore et al., 2018; Lee, 2020; Leonhard et al., 2020).

Few studies have experimentally tested the link between social media news exposure and knowledge acquisition. Bode (2016) found that participants could recall the type of political videos they watched on Facebook but struggled with details. Similarly, Feezell and Ortiz (2021) observed little factual learning after news scrolling, though they hypothesized that exposure might boost confidence without improving actual understanding, as earlier work on traditional media had shown (Park, 2001).

As far as we are aware, only two experimental studies have examined the relationship between news media exposure, perceived knowledge, and its discrepancy with actual knowledge (Anspach et al., 2019; Schäfer, 2020). Both experiments were implemented as between-subjects designs where participants were first exposed to a newsfeed and then asked about their perceived and factual knowledge. The topics of investigation were artificial sweeteners in one case and genetically modified foods in the other. The results indicated that participants who scrolled through many article previews had a significantly higher perceived knowledge that did not match their actual knowledge, compared with subjects who scrolled through only two headlines (Schäfer, 2020) or no news at all (Anspach et al., 2019). In both cases, a pre-test estimation of perceived knowledge was absent.

Another gap we identified in the literature is the limited variety of empirically tested topics employed as stimuli. Many of the studies mentioned above focused on political versus non-political information (Bode, 2016; Feezell & Ortiz, 2021; Weber & Koehler, 2017). Moreover, the research on the IoK has drawn a connection with extreme attitudes (Fernbach et al., 2019; Fernbach, Rogers, et al., 2013b, 2013a), suggesting that controversial and non-controversial topics might lead to different magnitudes of the effect. Following Park’s (2001) intuition, we believe that the key characteristic that might inflate perceived knowledge is the personal involvement of the individual, regardless of the topic being assessed.

In this study, we build on the existing literature connecting social media, perceived knowledge, and the IoK by directly testing the effect of news exposure. Two elements of novelty are worth noting. The first is the adoption of a pre-post within-subject design where participants’ assessments are recorded before and after exposure to a Facebook-like news feed. Secondly, we introduce a classification of topics based on personal involvement: As many political topics may be quite controversial for the general public, not all political topics are equally involving, and not all the controversial topics are strictly political. The preliminary screening to determine the experimental topics is described in the supplementary materials.

The present research was submitted and accepted as a Registered Report (10.24072/pci.rr.100986), meaning that the study design and analysis plan underwent peer review and received in-principle acceptance prior to data collection.

Our first hypothesis predicts the effect of exposure on perceived knowledge:

H_1_: Perceived knowledge of topics in the news feed will increase more than for topics not in the feed.

We also expect differences across levels of self-involvement:

H_2_: The effect of the news feed on perceived knowledge will be strongest in the high self-involvement group, followed by the medium group, and weakest in the low group.

A second set of hypotheses addresses the IoK, predicting that perceived and actual knowledge will not align:

H_3_: The discrepancy between perceived and factual knowledge will be positive and significantly different from zero.

H_4_: The IoK will be greater for topics present in the news feed than for those absent.

H_5_: The IoK will vary by self-involvement, with the strongest effect in the high group, followed by the medium, and weakest in the low group.

## Methods

### Sample size rationale

The estimation of the sample size of 800 subjects was based on budget constraints. This notwithstanding, we performed a series of power analyses for the perceived knowledge hypotheses (H_1_ and H_2_) based on a series of simulations of the experiment (https://osf.io/dc3ab/files/873wy). The simulations build on the sample size, an α of 5% (unidirectional), and a series of plausible values of the main variables, including the effect size (the increase in perceived knowledge) and the standard deviation of the effect size.

Given that the experiment was conducted at two time points, we also considered an attrition rate of 15% based on conservative estimates from a previous longitudinal study with a similar gap between sessions (Panizza et al., 2022). Based on this estimate, we recruited roughly 950 participants to ensure the minimum sample size of* N* = 800.

### Participants

A total of 828 participants (mean age = 48.5 years, *sd* = 15) took part in our study. The sample was a representative panel recruited through Bilendi, an online labour market platform. The only requirement to participate in the study was being Italian native speakers above 18 years old. Participants were remunerated according to Bilendi’s guidelines for online studies.

### Study design

This research protocol consists of a mixed design (between- and within-subjects), two-stage study composed by stimuli presentation and brief post-exposure questionnaires. Participants were randomly assigned to one of three experimental groups, characterized by the content of the newsfeed they scrolled through.

### Experimental protocol

The experiment was organized into two sessions. The first session collected self-reports and questionnaires, and it was the same for all participants. They were asked to estimate their knowledge about six topics. Topics varied by how personally involving they were perceived to be, as measured in a preliminary screening. In addition, participants were asked to assess how much they felt involved by each topic, and to express their attitude towards them. Afterwards, a psychometric assessment followed: scales were administrated to measure participants’ cognitive style, political orientation, and social media use. Finally, demographics information was collected.

The second session was scheduled two weeks after the first. Participants were randomly assigned to one of three experimental groups, characterized by the different content of the news headlines they were exposed to: low, medium, or highly self-involving group. We controlled whether randomization leads to unbalanced distributions of the psychometric variables (cognitive style, political position), and found no distortions.

**Fig. 1:**
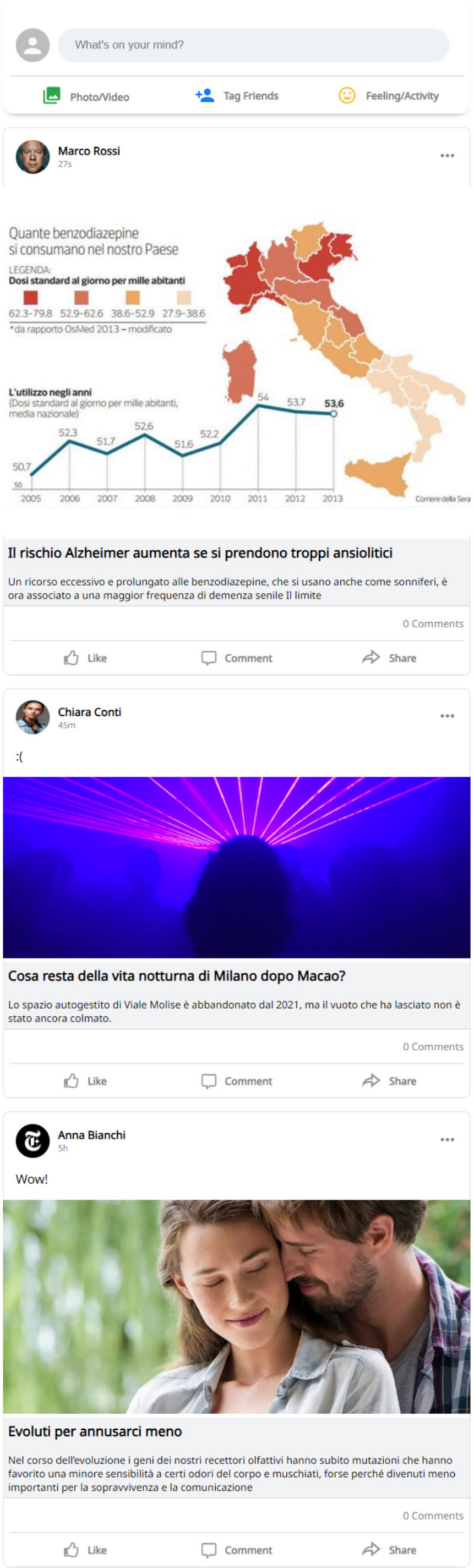
An example of newsfeed with three articles

Participants were redirected towards a mock social media news feed (Jagayat et al., 2021) that resembled that one of Facebook (see Fig. 1). There, they scrolled a series of news posts about the two topics assigned to their experimental group and a series of unrelated posts. The news headlines were composed by a title, an image, and a short description of the content. Users were able to react or comment under the news posts but they were not allowed to open the original articles. Posts in the news feed were displayed in random order.

After the exposure to the news feed, participants were asked again to fill up self-reports of perceived knowledge, self-involvement, and attitudes about all the six topics, not only those they were exposed to. Finally, their factual knowledge was measured with 10 True/False/Don’t Know statements for every subject to compute the illusion of knowledge.

### Manipulated variables

The manipulated variables were the content of the experimental stimuli for each group. The three groups are included as the three levels of the regression (Table 1).

**Table 1:** The experimental groups and the assigned topics

Low self-involvement	Medium self-involvement	High self-involvement
Feline immunodeficiency *I Promessi Sposi*	Anxiolytics Evolutionism	Abortion Climate change

### Measured variables and indices

- ***Perceived knowledge***. We measured perceived knowledge with one item for each of the six topics, asking participants “How much do you think you know about [this topic]?”, and they answered using a 0–100 visual analogue scale, going from 0 = *Nothing* to 100 = *Everything*. The score of perceived knowledge was computed as the participants’ evaluation/100, resulting in an index with a 0–1 range.
- ***Factual knowledge***. Participants’ actual knowledge was computed as the proportion of correct answers in the knowledge assessment at T2. For each topic, they read 10 statements, and for each statement they provided an answer among the options: True; False; I don’t know. Such assessment is thoroughly discussed in Appendix B. The score of factual knowledge was computed as the proportion of correct answers: number of correct answers/10, resulting in an index with a 0–1 range.
- ***Illusion of knowledge***. The perceived and the actual knowledge were standardized and combined to compute an index of illusion of knowledge. The index was calculated as the difference between the perceived knowledge at T_2_ and actual knowledge, that is the proportion of correct answers: IoK = perceived_T2_ – factual. For example, participants who scored 50 on perceived knowledge received a score of 0.5. If they had five correct answers, their actual knowledge score would be 0.5, resulting in an illusion of knowledge score of zero, as they accurately assessed their level of knowledge. This means that the Ki values range from 1 (i.e., the person reports maximum knowledge, but scores 0 on the knowledge test) to −1 (i.e., the person reports not having any knowledge on the topic but gives only correct answers on the knowledge test).

The full list of measured variables and the related exploratory hypotheses is available in the supplementary materials.

## Results

All the analyses were conducted using R Studio. The Analysis script and data can be found in the OSF folder at the following link (https://osf.io/dc3ab/files/osfstorage#). The complete summary of descriptives statistics and analyses are reported in the supplementary materials.

The contrast comparing the change in perceived knowledge from T_1_ to T_2_ between topics in the feed (exposed) and not seen in the feed (non-exposed) (H_1_). revealed an estimated difference of 0.73 (*SE* = 0.552), with a *z-*ratio of 1.316 and *p* = 0.188. This result suggests that the increase in perceived knowledge for topics in the feed was not significantly greater than the increase for topics not in the feed. We detected a significant increase in perceived knowledge about exposed topics from T_1_ to T_2_ (*p* = 0.017), although the direct pre- versus post-contrast was not preregistered.

To test the effect of self-involvement on perceived knowledge (H_2_) we computed a third-level contrast, testing for differences in the H_1_ contrast between topics of different levels of self-involvement. The test was not significant.

**Fig. 2:**
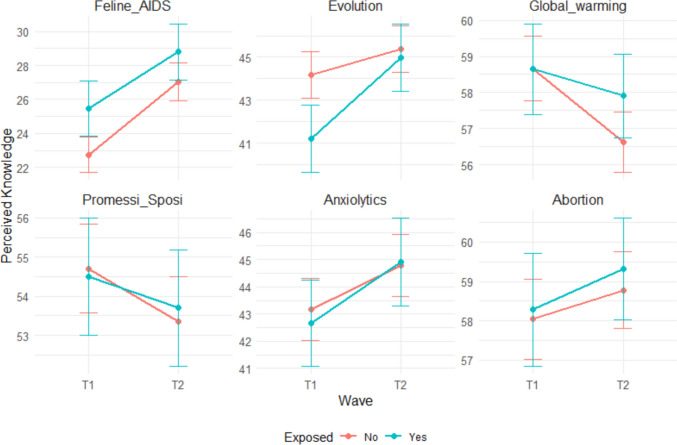
Descriptive plots of the results. The error bars represent the standard error. From left to right, the subplots depict the three experimental groups: low, medium, and high self-involvement. (Color figure online)

Thus, our prediction was not confirmed by the result: the self-involvement feature of the topics did not affect the shift in perceived knowledge Fig. 2.

The third hypothesis (H_3_) regarded the mere existence of an IoK effect. The median value of IoK was significantly higher than zero (*p* < 0.001) with differences across topics (see supplementary materials). Both tests of IoK differences due to exposure (H_4_) and due to exposure and level of self-involvement (H_5_) were not significant.

## Discussion

The present study aimed to test the influence of social media exposure on perceived knowledge about topics selected to elicit low to high self-involvement. While most of our hypotheses did not receive support from the data, the findings highlight several patterns worth further exploration.

Our first hypothesis regarding the mere effect of exposure was partially confirmed. While a pre-post comparison for seen topics revealed a significant increase, the difference-in-difference analysis did not yield significant results, implying that the observed increase could not be confidently distinguished from changes in the non-seen topics. Indeed, perceived knowledge of four out of six topics increased from T_1_ to T_2_, even though they were not included in the group’s stimuli. Disentangling this effect is challenging. One possibility is that we captured a test effect: simply being asked about a topic at T_1_ may have increased participants’ awareness or prompted them to seek information, leading to higher perceived knowledge at T_2_ regardless of our manipulation. Indeed, nearly all non-exposed topics showed increases over time, despite the two-week interval we introduced to reduce such anchoring effects.

Our second hypothesis examined whether differences in topic involvement influenced perceived knowledge shifts. Although the results were not statistically significant, an intriguing trend emerged that contrasted with our expectations: low- and medium-involvement topics appeared more likely to elicit changes in perceived knowledge. This observation aligns with social psychology literature. Perceived knowledge is considered one of the components of an attitude (Fabrigar et al., 2005), and stronger attitudes, typically associated with high involvement, are more resistant to change (Thomsen et al., 1995).

Consistent with our third hypothesis and with prior research, we expected participants to overestimate their knowledge, a prediction supported by our data. Interestingly, not all topics were equally susceptible to this effect. Notably, feline immunodeficiency and anxiolytics exhibited a reversed pattern, with participants underestimating their knowledge.

While the literature on metacognition has explored various domains, it remains unclear why some topics are more prone to the IoK than others. Interestingly, despite their apparent differences, the two topics share a connection to the healthcare domain and can be perceived as more technical than the others. Zeveney and Marsh (2016), who compared the magnitude of the IoK about devices versus mental disorders, have found that the latter were less susceptible to the phenomenon. In their discussion, the authors suggest that when knowledge is perceived as concentrated within a specific group of experts, individuals may be less prone to overestimate their own understanding. Thus, identifying themselves as non-experts (in our case, as non-healthcare professionals), our participants may have reported a lower degree of perceived knowledge compared with other topics.

Regarding topics that, instead, exhibited a significant IoK, two other explanations can be provided. High-involvement topics, such as abortion and climate change, likely prompted participants to positively assess their knowledge due to the personal importance of these issues. As confirmed in our data, self-involvement correlates with perceived knowledge, and, traditionally, is also associated to stronger attitudes (Thomsen et al., 1995). This pattern mirrors findings by Fernbach et al. (2019), who, in their study about GMOs, found that the discrepancy between perceived and actual knowledge increased with the extremity of the opposition to GMOs.

Two additional topics, the theory of evolution and *I Promessi Sposi*, also displayed significant IoK, an effect that may be explained through social desirability. An experiment ran by Gaviria and Corredor (2021) have investigated the role of social desirability of knowledge regarding historical events. The authors detected higher IoK associated with topics whose knowledge was rated as more socially desirable. In our case, evolution and *I Promessi Sposi* are standard components of the compulsory school curricula, from primary to high school. Thus, participants may have felt a sense of obligation to declare a familiarity not supported by real knowledge.

Finally, our fourth and fifth hypotheses predicted that the IoK would be influenced by social media exposure and by the condition. The hypotheses were not supported. These null results could be ascribed to a ceiling effect, where the IoK is too strong to be affected by our experimental manipulation. It could also be that the nature of social media exposure itself—characterized by frequent and repetitive interactions—may not have been adequately captured by our single-exposure design.

This result could be attributed to some limitations of the study, and to some intrinsic obstacles in conducting social media research. It is possible that our experimental manipulation was not strong enough to elicit an effect. However, it is worth noting that Schäfer (2020) observed a measurable effect using fewer Facebook posts (6) compared with our protocol (8). Furthermore, the null results could also be due to the limited sample quality, as many subjects failed attention and manipulation checks. If this is the case, passive scrolling alone may be insufficient to influence perceived knowledge. There is, indeed, some research regarding the differences between active and passive social media use (Verduyn et al., 2022). However, studies under this framework have primarily focused on emotional outcomes, leaving cognitive variables unexplored. Finally, it is difficult to interpret our findings in the context of previous research, as, to our knowledge, no prior studies have employed a within-subjects design to investigate the effects of mere exposure on perceived knowledge.

The evidence we gathered also underscores broader methodological challenges in studying social media and cognition. A standard experimental protocol relying on pre- and post-assessments with a single exposure may not fully capture the fragmented and sporadic nature of typical social media usage. Users often interact with these platforms in brief, dispersed sessions throughout the day for several days in a row. Future research should consider adopting designs that better mimic real-world engagement patterns to enhance ecological validity.

Furthermore, while null results are not direct evidence of no effect, it is also plausible that social media platforms are less influential in shaping public knowledge than previously assumed, and our findings reflect this reality. Recent research involving 35,000 social media users (Allcott et al., 2024) investigated the impact of Facebook deactivation on political knowledge, finding no significant effect on overall political knowledge. These outcomes suggest that the effects of social media platforms on knowledge and behavior may be less substantial than often presumed.

## Supplementary Information

Below is the link to the electronic supplementary material.

Supplementary file1 (DOCX 171 KB)

## Data Availability

The datasets generated during the current study are available in the OSF repository, together with the analyses code: https://osf.io/dc3ab/files/osfstorage#. All the materials (stimuli, questionnaires and translations, knowledge assessments) are available in the supplementary materials.
