# Supplementary material for: Scrolling to wisdom: The impact of social media news exposure on knowledge perception
Source: Psychon Bull Rev. 2025 Dec 10;33(1):11. doi: 10.3758/s13423-025-02786-3 (PMC12696030; doi:10.3758/s13423-025-02786-3)
Supplement: Supplementary file 1 — Supplementary file1 (DOCX 171 KB) [file 13423_2025_2786_MOESM1_ESM.docx]

## Scrolling to wisdom: the impact of social media news exposure on knowledge perception.

## **Supplementary materials**

[Table 1. Demographic information of the sample 2](#_Toc208841375)

[Attitudes and psychometric assessments 3](#_Toc208841376)

[Attention and manipulation checks 4](#_Toc208841377)

[Analysis plan 4](#_Toc208841378)

[Exploratory research questions 6](#_Toc208841379)

[Complete results 8](#_Toc208841380)

[Table 6. Descriptive statistics of perceived knowledge across topics and waves. 10](#_Toc208841381)

[Preliminary classification of topics 14](#_Toc208841382)

[Standardisation of knowledge assessment 15](#_Toc208841383)

[Stimuli 21](#_Toc208841384)

[Questionnaires and assessments 25](#_Toc208841385)

[References 29](#_Toc208841386)

| Table 1. Demographic information of the sample. | | |
| --- | --- | --- |
|  |  | *n* |
| Age | 18-34 | 183 |
|  | 35 – 44 | 149 |
|  | 45 – 54 | 191 |
|  | 55 – 64 | 168 |
|  | 65+ | 137 |
| Gender | Female | 398 |
|  | Male | 427 |
|  | Non-binary | 2 |
|  | Not declared | 1 |
| Highest educational attainment | Elementary school | 10 |
|  | Middle school | 61 |
|  | High school diploma | 423 |
|  | Bachelor’s degree | 105 |
|  | Master’s degree | 199 |
|  | PhD | 30 |
| Most used social media | Facebook | 425 |
|  | Instagram | 244 |
|  | TikTok | 40 |
|  | LinkedIn | 32 |
|  | Twitter/X | 25 |
|  | Twitch | 2 |
|  | Reddit | 1 |
|  | I don’t use social media | 46 |
|  | Other | 13 |
| Time spent on social media | Less than 10 minutes | 156 |
|  | About 1 hour | 321 |
|  | About 2 hours | 192 |
|  | About 3 hours | 84 |
|  | About 4 hours | 48 |
|  | More than 5 hours | 27 |

### Attitudes and psychometric assessments

Secondary variables were included to test exploratory analyses listed below. Each item of the selected scales was framed as follows: “How much do you agree with the following statements?”, and participants were asked to answer using a 0 – 100 scale going from 0 = *Totally disagree* to 100 = *Totally agree*. All the items are available in appendix D at the end of the document, and the assessment within the experimental protocol is described in figure 4.

The following three measures were taken only at T_1_:

- ***Cognitive style***. To capture participants’ cognitive style, we used the Rational-Experiential Inventory short (REI-10), a combination of 5 items taken from the Need for Cognition (Cacioppo & Petty, 1982) and 5 items from the Faith in Intuition (Epstein et al., 1996). This scale was designed to assess preferences for information processing, and to distinguish between an analytical versus affective approaches.
- ***Cultural worldview***. To assess the political view of the participants, we used the short version of the Cultural Cognition Worldview Scale (CCWS) (Kahan, 2012) that allows us to measure the predispositions onto two sub-scales: 6 items to identify the position on the individualism/communitarianism axis, and 6 items for hierarchy/egalitarianism.
- ***Social media use***. To estimate how intense is participants’ use of social media, we adapted The Multidimensional Facebook Intensity Scale (Orosz et al., 2016). The scale captures four main facets of Facebook use: boredom, self-expression, over-use, and persistence. We first will ask participants’ which is their most used social media, and then use their answer to articulate the questions.

The following three measures were recorded both at T_1_ and T_2_:

- ***Opinion extremity***.^^[[1]](#footnote-1)^^ For each topic, participants were requested to express their opinion. We asked them to answer to two items per theme: one framed in a positive valence, e.g. “I think we should spread more information about Evolutionism”, and one framed with the opposite valence, e.g. “I think that schools are spending too much time teaching Evolutionism”, computed with a reverse score. All the items were formulated taking inspiration from the common formulas used to measure explicit attitudes in health and social psychology (Eldredge et al., 2016). The extremity of the opinion was calculated as the distance of the result from 50, the centre of the scale.
- ***Self-involvement***. To record participant’s self-involvement, they answered to two items regarding their perceived involvement and their willingness to discuss the topic. The formulation of this question was the same as the one used in the pre-test. The self-involvement score was computed as the average between the two items.
- ***Intellectual humility***. We measured participants’ intellectual humility using the General Intellectual Humility Scale (Leary et al., 2017).

### Attention and manipulation checks

Some additional control questions were administrated to check whether subjects had paid attention to the experimental stimuli and environment. As a robustness check, we repeated all the pre-registered analyses excluding those participants who failed all the attention and manipulation checks.

- ***Attention check****.* Within the administered questionnaire for psychometric assessment, we included items aimed to test whether the participant is actually reading the questions or not, like: “Please answer “Totally disagree” to this question”. Similar checks were included in the knowledge tests.

At T1 participants were asked to leave the slider bar at the center (indicating the value of 50). We considered the attention check failed when participants set the bar in a position lower 35 and higher than 65.

- ***Manipulation check****.* After scrolling through the social media feed, participants were asked to recall the news posts present in the news feed. This helps ensure that participants have been actively processing and retaining information. In particular, we asked them if they remembered to have seen news about two topics, by selecting the stimuli among six randomly selected news (one for each topic). The assessment of participants’ score worked as follows: for each correct answer, they got 1 point; for each wrong answer they had 1 point subtracted. We considered the manipulation check “passed” when participants had a score of 1 or more.

### Analysis plan

All analyses are conducted using a 5% significance threshold. Multiple comparisons are corrected using the false discovery rate method.

H_pre_: For each topic, there is no significant difference between the preliminary screening self-involvement ratings and the ratings before exposure.

To evaluate H_0_, we conducted an equivalence test (Two One-Sided Tests or TOST) for each topic. Specifically, we examined whether the self-involvement mean of each topic falls within a specified range of +/- 10 points from the mean value in the preliminary screening.

This analysis serves to ensure that none of the topics are inadvertently misclassified within an incorrect category, safeguarding the integrity of our study.

This group of hypotheses concern the dependent variable perceived knowledge. For a sample of the analysis pipeline, please refer to the R script the simulates the analyses for computing a power analysis ([**Script_PA**](https://osf.io/2e65h)).

The first experimental hypothesis predicts the effect of exposure on perceived knowledge:

H_1_: Perceived knowledge of topics in the news feed will increase more than perceived knowledge of topics not in the news feed.

**Figure SEQ Figure \* ARABIC 1**. The solid line follows the expected trend of the variable perceived knowledge for exposed topics, whereas the dashed line represents the expected trend of non-exposed topics. The black dots indicate the average of perceived knowledge for exposed topics. We predict no change in non-exposed topics between T1 and T2.

To test for H_1_, we computed a difference in differences contrast between perceived knowledge ratings of topics inside/outside the news feed, at T_1_ and at T_2_:

$$\beta_{feed \times T2}-\beta_{feed \times T1}>\beta_{no feed \times T2}-\beta_{no feed \times T1}$$

We then performed six post-hoc equivalence tests, one for each topic, to confirm that the perceived knowledge of participants at T_1_ did not differ between the three experimental groups (e.g., one group displaying higher perceived knowledge about one topic than the other two groups). This ensured that any effects of exposure did not load on pre-existing differences among groups. In particular, we checked whether the average perceived knowledge of a topic fell in the same +/- 10 points range in each experimental group.

H_1bis_: Perceived knowledge at T_0_ will not differ significantly between exposed and non-exposed topics.

H_2_: The effect of the news feed on perceived knowledge will be greater in the high self-involvement group compared to the low and medium self-involvement groups,and in the medium self-involvement group compared to the low self-involvement group.

To test for H_2_, we computed a third-level contrast, testing for differences in the H_1_ contrast between topics of different levels of self-involvement, namely:

H_2A_: $\left[ \left( \beta_{feed \times T2}-\beta_{feed \times T1} \right)-\left( \beta_{no feed \times T2}-\beta_{no feed \times T1} \right) \right]_{high}>$

$$\left[ \left( \beta_{feed \times T2}-\beta_{feed \times T1} \right)-\left( \beta_{no feed \times T2}-\beta_{no feed \times T1} \right) \right]_{med}$$

H_2B_: $\left[ \left( \beta_{feed \times T2}-\beta_{feed \times T1} \right)-\left( \beta_{no feed \times T2}-\beta_{no feed \times T1} \right) \right]_{high}>$

$$\left[ \left( \beta_{feed \times T2}-\beta_{feed \times T1} \right)-\left( \beta_{no feed \times T2}-\beta_{no feed \times T1} \right) \right]_{low}$$

H_2C_: $\left[ \left( \beta_{feed \times T2}-\beta_{feed \times T1} \right)-\left( \beta_{no feed \times T2}-\beta_{no feed \times T1} \right) \right]_{med}>$

$$\left[ \left( \beta_{feed \times T2}-\beta_{feed \times T1} \right)-\left( \beta_{no feed \times T2}-\beta_{no feed \times T1} \right) \right]_{low}$$

Tests of H_1_ and H_2_ we employed a mixed-effects linear regression with self-reported perceived knowledge as dependent variable, and the following independent variables:

- **Topic** involvement (3 levels: FIV and PP as low, ANS and DAR as medium, GW and IVG as high). Given that the topics selected differed in terms of perceived knowledge in the preliminary screening, it is likely that this variable would significantly predict perceived knowledge on its own, but we do not propose a specific hypothesis on this relation.^^[[2]](#footnote-2)^^
- **Time** of reporting (0 if T_1_, 1 if T_2_). Time should not be a significant predictor of perceived knowledge if not in interaction with the news feed. In other words, if a topic is not covered in the news feed, we do not expect any systematic change in perceived knowledge between T_1_ and T_2_.
- Presence of the topic in the **news feed** (0 if present, 1 if absent). Topic exposure should not be a significant predictor of perceived knowledge at T_1_ (since exposure did not happen yet, in line with H_1bis_), but only at T_2_. Thus, the effect of exposure should be significant only in interaction with time.
- **Interaction** between **time** and **news feed** exposure, required for the second-level contrasts (difference in differences) that test for H_1_.
- **Interaction** between **time** and **topic**, which we expect to be non-significant, unless a topic is covered in the news cycles occurring between T_1_ and T_2_.
- **Interaction** between **news feed** exposure and topic, which we expect to be non-significant.
- **Interaction** between **time, news feed** exposure, and **topic**, required for the third-level contrasts that test for H_2_.

The regression included by-participant random intercepts and random slopes for time, news feed and topic.

H_3_: The discrepancy between the reported perceived knowledge and the measured actual knowledge will be positive and significantly different from zero.

H_4_: The illusion of knowledge will be greater for topics present in the news feed compared to topics not present in the news feed.

H_5_: The effect of the news feed on the illusion of knowledge will be greater in the high self-involvement group compared to the low and medium self-involvement groups, and in the medium self-involvement group compared to the low self-involvement group.

Hypotheses H_3_, H_4_ and H_5_ was tested using a mixed-effects linear regression with illusion of knowledge as predicted variable with independent variables topic, news feed and their interaction (time is not included as the illusion of knowledge is measured only at time T_2_), and with by-participant random intercepts and slopes for time and news feed exposure. H_3_ was tested with the contrast $\beta_{no feed}>0$, where $\beta_{no feed}$ is the aggregated coefficient of all topics in the absence of the news feed. H_4_ was tested with the contrast $\beta_{feed}>\beta_{no feed}$, and H_5_ was tested with the second-level contrasts:

H_5A_: $\left( \beta_{feed}-\beta_{no feed} \right)_{high}>\left( \beta_{feed}-\beta_{no feed} \right)_{med}$

H_5B_: $\left( \beta_{feed}-\beta_{no feed} \right)_{high}>\left( \beta_{feed}-\beta_{no feed} \right)_{low}$

H_5C_: $\left( \beta_{feed}-\beta_{no feed} \right)_{med}>\left( \beta_{feed}-\beta_{no feed} \right)_{low}$

### Exploratory research questions

In the following section, we list and briefly describe the effects that we aimed to explore with combinations of the main and secondary variables, even if they are not part of the experimental hypotheses.

- Informed by the preliminary screening we conducted (see appendix A), we explored at T_1_ whether assessments of perceived knowledge and self-involvement reported by the subjects are correlated. We ran 6 correlation tests, one for each topic.
- We conducted six equivalence tests, one for each topic, to compare perceived knowledge of participants at T_1_ with the evaluations obtained from the preliminary screening.
- For each topic, we measured whether strength of attitude towards the topic correlates with self-involvement at T_1_.
- We examined potential gender differences in the magnitude of the illusion of knowledge by adding gender and its interactions with the other variables as covariates to test H_1_.
- Although participants were not able to open the news articles, we recorded their attempts to click on the links. We investigated possible correlations between click rates on the article and both perceived knowledge and illusion of knowledge at T_2_.
- Toplak et al. (2014) found that a reflective cognitive style is associated with reduced biases and more accurate judgement. We tested whether the score of reflective thinking predicts the illusion of knowledge by adding cognitive style as a covariate in the linear regression testing the effect of news feed exposure and self-involvement on the illusion of knowledge. We similarly added social media use as an additional covariate of illusion of knowledge.
- The literature about confirmation bias, the propensity to seek and interpret information to confirm rather than dis-confirm our prior beliefs, suggests that an attitude towards a topic can be strengthened after being exposed to arguments and statements about that matter (Lord et al., 1979). Following H_1_, we then tested an effect of news feed exposure on the strength of attitudes.
- We tested whether it is possible to detect a shift in other variables (self-involvement and intellectual humility) between T1 and T2.

### Complete results

All the analyses were conducted using R Studio. The Analysis script can be found in the OSF folder at the following link: [https://osf.io/dc3ab/files/osfstorage#](https://osf.io/dc3ab/files/osfstorage)

As a first analysis (H_pre_), we compared the self-involvement assessments at T_1_ with the corresponding assessments recorded during the pre-test conducted on a different sample but from the same population.

As shown in Table 1, the self-involvement assessment of the experimental sample was overall more moderate as compared to that of the Pre-test. In particular, participants recruited for the experiment were more involved in low-involvement topics (see upper bound insignificance for the two low-involvement topics), and less involved in the high-involvement topics (see lower bound insignificance for the two high-involvement topics).

Despite this negative finding, these results did not affect the group organization. First, the negative result does not affect the hypotheses (H_1_, H_3_ and H_4_) regarding the mere exposure and, furthermore the relative ranking of topics in terms of self-involvement remained the same, therefore we do not expect results too far from the ones hypothesized with the three categorizations. I Promessi Sposi stands out for its closeness to the mean self-involvement of the medium involvement group.

| **Table 2.** The results of the TOSTs performed to compare pre-test measures with T_1_ measures. | | | | | |
| --- | --- | --- | --- | --- | --- |
| **Topic** | **Pre-test**  mean(*sd*) | **T_1_**  mean(*sd*) | **Upper bound**  *p* | **Lower bound**  *p* | **T-Test**  *p* |
| Feline immunodeficiency | 17.3(*24.9*) | 28.4(*27.1*) | 0.643 | <.001 | <.001 |
|  |  |  |  |  |  |
| I Promessi Sposi | 22.6(*23.8*) | 37.9(*28.1*) | 0.964 | <.001 | <.001 |
| Evolution | 40.3(*27.8*) | 40.2(*26.4*) | <.001 | <.001 | 0.970 |
| Anxyolitics | 39.7(*28.4*) | 38.9(*27.8*) | <.001 | <.001 | 0.797 |
| Global Warming | 74.1(*23.6*) | 60.5(*25.9*) | <.001 | 0.910 | <.001 |
| Abortion | 67.6(*30.2*) | 52.8(*27.7*) | <.001 | 0.950 | <.001 |

*H_1_ and H_2_, the effect of exposure on perceived knowledge*

We then tested the effect of the mere exposure on perceived knowledge (H_1_). The contrast comparing the change in perceived knowledge from T_1_ to T_2_ between topics in the feed (exposed) and not seen in the feed (non-exposed) revealed an estimated difference of 0.73 (SE = 0.552), with a z-ratio of 1.316 and a *p*-value of 0.188. This result suggests that the increase in perceived knowledge for topics in the feed was not significantly greater than the increase for topics not in the feed.

We furthermore detected a significant increase in perceived knowledge about exposed topics from T_1_ to T_2_ (see table 3), although the direct pre- vs post- contrast was not preregistered.

| **Table 3.** Results of the difference between T_1_ and T_2_ for exposed and non-exposed topics. | | | |
| --- | --- | --- | --- |
|  | **Mean difference**  T_2_ – T_1_ | **SE** | ***p*** |
| Non-exposed | 0.74 | 0.53 | 0.157 |
| Exposed | 1.47 | 0.62 | 0.017 |
| (T_2_ – T_1_ Exp) – (T_2_ – T_1_ Non Exp) | 0.73 | 0.552 | 0.188 |

We furthermore performed six post-hoc equivalence tests, one for each topic, to confirm that the perceived knowledge of participants at T_1_ did not differ between the three experimental groups (H_1bis_, e.g., one group displaying higher perceived knowledge about one topic than the other two groups). This ensured that any effects of exposure did not load on pre-existing differences among groups. In particular, we checked whether the average perceived knowledge of a topic fell within the same +/- 10 points range in each experimental group (see Table 4).

| **Table 4.** The results of the TOSTs comparing perceived knowledge for all topics across all groups. The shading indicates the experimental group (three levels). | | | | |
| --- | --- | --- | --- | --- |
| **Self-involvement** | **Topic** | **Lower bound *p*** | **Upper bound *p*** | **T-Test *p*** |
| Low | *Feline immunodeficiency* | <.001 | <.001 | 0.14 |
|  | *I Promessi Sposi* | <.001 | <.001 | 0.91 |
| Medium | *Evolution* | <.001 | <.001 | 0.11 |
|  | *Anxyolitics* | <.001 | <.001 | 0.8 |
| High | *Global Warming* | <.001 | <.001 | 0.99 |
|  | *Abortion* | <.001 | <.001 | 0.89 |

All the performed TOST tests resulted in significant results, suggesting that each group had an equal perceived knowledge score before any exposure. This result also ensures that the participants were successfully randomized in the experimental groups.

To test the effect of self-involvement on perceived knowledge (H_2_) we computed a third-level contrast, testing for differences in the H_1_ contrast between topics of different levels of self-involvement. The test was not significant (see table 5).

| **Table 5.** The computed contrasts for H_2_. | | | | |
| --- | --- | --- | --- | --- |
| **Contrast** | **Estimate** | **SE** | **Z** | ***p*** |
| High vs low | 0.484 | 1.69 | 0.286 | 0.77 |
| High vs Medium | -1.942 | 1.69 | -1.149 | 0.25 |
| Medium vs low | 2.427 | 1.69 | 1.438 | 0.15 |

Thus, our prediction was not confirmed by the result: the self-involvement feature of the topics did not affect the shift in perceived knowledge. The results are summarized in table 6.

| Table 6. Descriptive statistics of perceived knowledge across topics and waves.  Average rating of each topic at T1 and T2, divided by whether the topic was present in the news feed or not. The last column indicates the difference between averages. | | | | | |
| --- | --- | --- | --- | --- | --- |
|  | Topic | In the newsfeed | Perceived knowledge at T1 | Perceived knowledge at T2 | T2 – T1 |
| High involvement | Abortion | No | 58.04 | 58.8 | 0.76 |
|  |  | Yes | 58.3 | 59.3 | 1 |
|  | Global warming | No | 58.66 | 56.63 | -2.03 |
|  |  | Yes | 58.64 | 57.9 | -0.74 |
| Medium involvement | Anxiolytics | No | 43.17 | 44.8 | 1.63 |
|  |  | Yes | 42.67 | 44.9 | 2.23 |
|  | Evolution | No | 44.2 | 45.38 | 1.18 |
|  |  | Yes | 41.2 | 44.98 | 3.78 |
| Low involvement | Feline AIDS | No | 22.74 | 27.04 | 4.3 |
|  |  | Yes | 25.5 | 28.8 | 3.3 |
|  | *I Promessi Sposi* | No | 54.71 | 53.36 | -1.35 |
|  |  | Yes | 54.5 | 53.7 | -0.8 |

*H_3_: the illusion of knowledge effect*

The third hypothesis (H_3_) regarded the mere existence of an illusion of knowledge effect. The median value of illusion of knowledge was significantly higher than zero (*p* < .001). Further tests were computed for each topic, as reported in table 7.

| **Table 7.** Descriptive statistics of the illusion of knowledge score. The first two columns represents means. The knowledge score has been normalized to be compared to the perceived knowledge assessment that was recorded on a 0 – 100 scale. | | | | | | |
| --- | --- | --- | --- | --- | --- | --- |
| **Topic** | **Knowledge score** | **Perceived knowledge at T_1_** | **Illusion of knowledge**  ***mean*** | **Illusion of knowledge 95% CI** | **Illusion of knowledge**  ***median*** | ***p*** |
| Feline immunodeficiency | 26.05 | 23.66 | -2.39 | -4.5; -0.274 | 1 | 0.978 |
| I Promessi Sposi | 44.89 | 54.64 | 9.75 | 7.73; 11.76 | 10 | < .001 |
| Evolution | 28.8 | 44.18 | 14.37 | 12.54; 16.2 | 14 | < .001 |
| Anxiolytics | 44.20 | 43 | -1.21 | 0.91; -3.32 | -1 | 0.924 |
| Global Warming | 40.18 | 58.66 | 18.47 | 16.79; 20.16 | 19 | < .001 |
| Abortion | 36.55 | 58.17 | 23.39 | 19.75; 23.39 | 22 | < .001 |

The results point out that even if, in general, participants significantly overestimated their knowledge, the overall effect was driven by a subset of topics, suggesting a differential influence of this phenomenon based on the topic. In particular, participants did not overestimate their knowledge about feline immunodeficiency and anxiolytics.

*The relationship between the illusion of knowledge and social media use (exploratory)*

In support of our 4^th^ and 5^th^ hypotheses we ran an exploratory test to measure the correlation between the illusion of knowledge score and participants’ self-reported social media consumption, measured with the corresponding scale (Orosz et al., 2016). We detected a significant correlation (*p* < .001) between social media use and the illusion of knowledge score, suggesting a relationship between the investigated variables.

*H_4_ and H_5_, the illusion of knowledge across groups*

Both tests of illusion of knowledge differences due to exposure (H_4_) and due to exposure and level of self-involvement (H_5_) were not significant (see table 8).

| **Table 8.** The resulting contrasts for H_4_ and H_5_. | | | | | |
| --- | --- | --- | --- | --- | --- |
|  | **Contrast** | **Estimate** | **SE** | **Z** | ***p*** |
| **H_4_** | Exposed vs non exposed | 0.0122 | 0.0652 | 0.188 | 0.85 |
| **H_5_** | High vs low involvement | -0.1898 | 0.1375 | -1.381 | 0.1674 |
|  | High vs medium involvement | -0.1772 | 0.1381 | -1.283 | 0.1995 |
|  | Medium vs low involvement | -0.0126 | 0.1374 | -0.092 | 0.9267 |

Thus, although we indeed detected the illusion of knowledge in our sample (H_3_), the phenomenon was not affected by the experimental manipulation.

*Exclusion of participants who failed manipulation and attention checks*

As preregistered, we repeated all the analyses excluding those participants who failed all the attention and manipulation checks.

The check filter was applied as follow: first, we included only the participants who passed the attention check at T_1_ (*n* = 739); from this subsample, we filtered those who also passed the attention check at T_2_ (*n* = 691). Finally, we filtered out the participants who failed the manipulation check, i.e. who failed to recognize the news present in their newsfeed. The total number of participants was therefore 394. We then repeated the analyses on two subsamples: first, on the 691 participants who passed the attention checks, and second on the 394 who passed both attention and manipulation checks. The results are reported in table 9.

| **Table 9.** The analyses repeated on the subsamples who passed the attention check (n = 691), and who passed both attention and manipulation checks (n = 394) | | | | | | | |
| --- | --- | --- | --- | --- | --- | --- | --- |
|  | | **Contrast** | **Estimate** | **SE** | **Z/t** | ***p*** | |
| **H_1_** | N = 691 | T_2_ – T_1_ (Exposed) | 1.376 | 0.638 | 2.156 | 0.0311 | * |
|  |  | (T_2_ – T_1_ Exp) – (T_2_ – T_1_ Non Exp) | 0.853 | 0.598 | 1.425 | 0.1540 |  |
|  | N = 394 | T_2_ – T_1_ (Exposed) | 1.847 | 0.846 | 2.185 | 0.0289 | * |
|  |  | (T_2_ – T_1_ Exp) – (T_2_ – T_1_ Non Exp) | 1.52 | 0.787 | 1.933 | 0.0532 |  |
| **H_2_** | N = 691 | High vs Low | –1.320 | 1.800 | –0.729 | 0.466 |  |
|  |  | High vs medium | 1.760 | 1.800 | 0.975 | 0.330 |  |
|  |  | Medium vs low | –3.07 | 1.800 | –1.709 | 0.087 |  |
|  | N = 394 | High vs Low | –1.683 | 2.380 | –0.708 | 0.479 |  |
|  |  | High vs medium | 0.981 | 2.390 | 0.411 | 0.681 |  |
|  |  | Medium vs low | –2.664 | 2.360 | –1.130 | 0.258 |  |
| **H_3_** | N = 691 | Illusion of knowledge vs 0 |  |  |  | < 0.000 | *** |
|  | N = 394 | Illusion of knowledge vs 0 |  |  |  | < 0.000 | *** |
| **H_4_** | N = 691 | Exposed vs non exposed | -0.121 | 0.71 | -0.170 | 0.57 |  |
|  | N = 394 | Exposed vs non exposed | -1.09 | 0.94 | -1.16 | 0.88 |  |
| **H_5_** | N = 691 | High vs low involvement | 0.46 | 2.21 | 0.208 | 0.83 |  |
|  |  | High vs medium involvement | -1.89 | 2.21 | -8.54 | 0.4 |  |
|  |  | Medium vs low involvement | 2.35 | 2.21 | 1.065 | 0.29 |  |
|  | N = 394 | High vs low involvement | 2.25 | 2.9 | 0.78 | 0.44 |  |
|  |  | High vs medium involvement | -1.92 | 2.9 | -0.7 | 0.51 |  |
|  |  | Medium vs low involvement | 4.17 | 2.9 | 1.45 | 0.15 |  |

This further examination, although lacking statistical power, indicates that, when participants are correctly engaged in the experiment, the results might go in the predicted direction regarding the mere effect of social media exposure. We could not, however, detect a significant shift in the illusion of knowledge, a psychological effect that, in our data, persists regardless of any manipulation.

#### Exploratory research questions

Our first exploratory hypothesis regarded the correlation between perceived knowledge and self-involvement, to replicate the finding of our pre-test. This relation was confirmed by the experimental data. Prior to any manipulation, at T_1_, perceived knowledge and self-involvement correlated with *r* = 0.623 (*p* < .001). Across topics the analyses yield significance (*p* <.001), varying in different degrees of *r*, but displaying a consistent value above 0.5. See table 10.

| **Table 10.** Correlations between self-involvement and perceived knowledge for each topic, at T_1_. | | |
| --- | --- | --- |
|  | **Pearson’s correlations** | |
|  | ***r*** | ***p*** |
| Global warming | 0.569 | <.001 |
| Abortion | 0.533 | <.001 |
| Anxiolytics | 0.635 | <.001 |
| Evolution | 0.598 | <.001 |
| I Promessi Sposi | 0.531 | <.001 |
| Feline Immunodeficiency | 0.652 | <.001 |

This result seems to suggest that the more someone feels involved regarding a subject, the higher is their perception of being knowledgeable about it.

To investigate the role of cognitive style, we first computed a simple correlation test between the analytical score obtained by the Need for Cognition items and the index of illusion of knowledge. The two variables resulted significantly and negatively correlated, even though with a small effect (*r* = -0.054, *p* < .001). Conversely, the intuition score was instead found to be positively correlated with the illusion of knowledge (*r* = 0.064, *p* <.001). Therefore, participants with a reflective cognitive style were less susceptible to the illusion of knowledge, whereas participants with a more intuitive cognitive style were a little more.

We additionally tested whether it was possible to detect a significant increase in the intellectual humility score between T_1_ and T_2_. We therefore performed a Wilcoxon signed-rank test which demonstrated a significant increase in the variable of interest (z = 2.743, *p* = .003). This result suggests that being asked to answer a series of questions, and likely facing the limits of one’s knowledge, might highlight such limitations, making them more accessible and thus increase temporarily intellectual humility.

We furthermore looked for gender differences in the illusion of knowledge, but it was not possible to identify significant differences nor trends. Males and females displayed the same pattern of bias, that is a significant illusion of knowledge regarding all the topics except anxiolytics and feline immunodeficiency. The latter was furthermore the topic displaying the biggest difference between males’ illusion of knowledge (m = -0.066) and females’ (m = -0.399), in the sense that females underestimated their knowledge to a larger extent. The difference was however not statistically significant (*p* = 0.123).

*Hypotheses regarding opinion extremity*

Finally, we proposed two exploratory hypotheses about opinion extremity.

Our first hypothesis aimed to investigate whether self-involvement in a topic correlates with the opinion extremity about topic-specific beliefs. The Pearson’s Correlation was significant (*r* = 0.209, *p* < .001), showing that the more a subject is involved in a topic, the more they will be distant from a neutral opinion. Any contrast comparing pre and post measures of opinion extremity was found non-significant.

### Preliminary classification of topics

A preliminary study has been conducted to select the content of the experimental stimuli. A total of 100 participants has been recruited through Prolific and asked to participate in a survey. The sample was balanced for gender.

The survey requested them to evaluate a selection of thirty topics on the dimensions of: emotional involvement, willingness to discuss the topic, and perceived knowledge. The topics were taken from many different domains, like science, politics, literature, health, law, ethics. All the themes were selected to be tested as objectively as possible in a knowledge test, with little room for partial or ideological interpretations.

**Figure 1**. The scatter plot showing the medians of perceived knowledge and self-involvement.


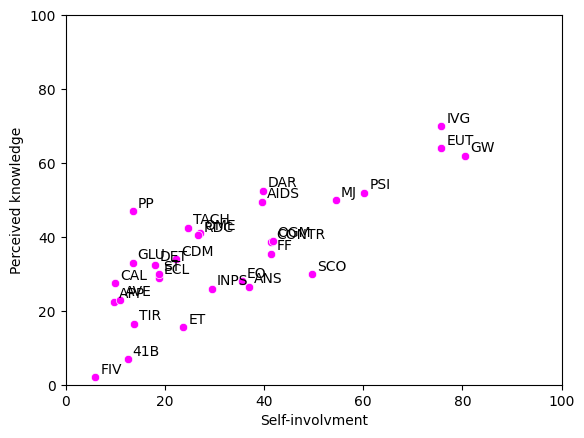


Given the strong correlation between emotional involvement and willingness to discuss (Cronbach’s alfa = 0.88), we decided to combine the two measures into the variable self-involvement, which informed our decision for the stimuli selection: the medians of perceived knowledge and self-involvement were used to classify the topics (fig 1).

We selected six topics, two with low, two with medium, and two with high self-involvement. The two topics for each level were selected to differ as much as possible on the dimension of perceived knowledge, in order to de-correlate as much as possible the two variables. The selection process resulted in the following topics:

- Feline immunodeficiency (FIV) and I Promessi Sposi (PP) classified as having low self-involvement;
- Evolutionism (DAR) and Anxyiolitics (ANS) as medium self-involvement;
- Global warming (GW) and abortion (IVG), as high self-involvement;

The selected topics were then included in the different news feeds in the form of news articles.

### Standardisation of knowledge assessment

For all the six selected topics, we created a scale of ten questions to test participants’ knowledge of each topic. The set of ten questions derived from an original list of about 20 questions per topic taken and adapted from the literature or based on online scientific and news materials.

The original list of questions was administered to a sample (n = 100) of participants recruited on the online platform Prolific. The sample consisted of Italian respondents and was balanced for gender. For each statement, participants could select an option among True / False / I don’t know.

The ability of respondents to answer the questions informed the final selection of items. For each topic, we first identified the questions with a high discrimination index, that is those items that were often correctly answered by the best-performing participants (the top 27%), and, at the same time, often missed by the worse-performing participants (the bottom 27%). When the discrimination index was comparable among items, qualitative considerations guided the final choice, for example: proportion of true and false statements, similarities among items, proportions of correct answers, and so on.

The final scales are the following:

| Cambiamenti climatici | | Climate change (GW) |
| --- | --- | --- |
| 1 | L’anidride carbonica (CO2) è un gas a effetto serra | Carbon dioxide (CO2) is a greenhouse gas |
| 2 | Il buco dell’ozono è la causa principale dell’effetto serra | The hole in the ozone layer is the main cause of the greenhouse effect |
| 3 | Nelle stesse quantità, la CO2 è più dannosa per il clima del metano | In the same quantities, CO2 is more harmful to the climate than methane |
| 4 | Per i prossimi decenni, la maggior parte della comunità scientifica si aspetta che il clima cambi in modo uniforme in tutto il mondo | For the next few decades, most of the scientific community expects the climate to change uniformly across the world |
| 5 | L’accordo di Parigi del 2015 segna l’impegno di 195 nazioni a mantenere l'aumento della temperatura media mondiale al di sotto di 2 °C rispetto ai livelli preindustriali | The 2015 Paris Agreement marks commitment by 195 nations to keep global average temperature increase below 2°C above pre-industrial levels |
| 6 | I cambiamenti climatici provocheranno un aumento dei casi di cancro | Climate change will cause an increase in cancer cases |
| 7 | Più del 10% della comunità scientifica nega la responsabilità umana nel riscaldamento globale | More than 10% of the scientific community denies human responsibility for global warming |
| 8 | Più della metà degli scienziati che sono scettici sul cambiamento climatico sono specializzati in climatologia | More than half of the scientists who are climate change skeptics specialize in climatology |
| 9 | Gli scienziati ritengono che i cambiamenti climatici aumenteranno il rischio di epidemie di infezioni virali | Scientists believe that climate change will increase the risk of an epidemic of viral infections |
| 10 | Il trasporto aereo è uno tra i settori più inquinanti in termini di emissioni di gas serra | Air transport is one of the most polluting sectors in terms of greenhouse gas emissions |

| Aborto | | Abortion (IVG) |
| --- | --- | --- |
| 1 | L’assunzione del farmaco per l’aborto farmacologico avviene tramite iniezione | The drug for medical abortion is taken by injection |
| 2 | I farmaci utilizzati per l’aborto farmacologico possono essere utilizzati anche per coadiuvare il completamento di un aborto spontaneo | Drugs used for medical abortion can also be used to help complete a miscarriage |
| 3 | In Italia è ammessa l’interruzione volontaria di gravidanza entro 90 giorni dal concepimento | In Italy, voluntary termination of pregnancy is permitted within 90 days of conception |
| 4 | L’attuale legge che regolamenta l’interruzione volontaria di gravidanza è stata approvata alla fine degli anni ‘70 | The current law regulating the voluntary termination of pregnancy was approved in the late 1970s |
| 5 | Aborto farmacologico e aborto chirurgico hanno lo stesso limite temporale, ovvero si può ricorrere all’uno o all’altro con le stesse tempistiche | Pharmacological abortion and surgical abortion have the same time limit, i.e. one or the other can be used with the same timing |
| 6 | Prima dell’approvazione dell’attuale legge sull’aborto, una donna che interrompeva volontariamente la gravidanza poteva essere punita con la reclusione in carcere. | Before the current abortion law was passed, a woman who voluntarily terminated her pregnancy could be punished with imprisonment |
| 7 | Agli inizi degli anni ‘80 un referendum proposto dal Partito Radicale propose di facilitare le procedure di accesso all’aborto | In the early 1980s, a referendum proposed by the Radical Party proposed facilitating the procedures for access to abortion |
| 8 | L’aborto si definisce terapeutico quando viene eseguito al fine di preservare la salute della madre | Abortion is defined as therapeutic when it is performed in order to preserve the health of the mother |
| 9 | La fertilità risulta compromessa per un certo periodo successivo all’aborto chirurgico | Fertility is impaired for a certain period following the surgical abortion |
| 10 | Nella fase di espulsione dell'embrione a seguito dell’intervento di aborto, il personale medico è obbligato a fornire assistenza sanitaria, anche se obiettore di coscienza | In the phase of expulsion of the embryo following the abortion, medical personnel are obliged to provide health care, even if they are conscientious objectors |

| I Promessi Sposi (PP) | | |
| --- | --- | --- |
| 1 | I bravi sono due fedeli servitori dell’Innominato | The *bravi* are two faithful servants of the Unnamed |
| 2 | Don Abbondio è un personaggio contraddistinto da una spiccata devozione religiosa | Don Abbondio is a character characterized by a marked religious devotion |
| 3 | Il primo personaggio a comparire nel romanzo è Padre Cristoforo | The first character to appear in the novel is Father Cristoforo |
| 4 | Perpetua è la domestica di Don Rodrigo | Perpetua is Don Rodrigo's maid |
| 5 | Manzoni dice di aver tratto le vicende raccontate nel romanzo da un manoscritto di autore anonimo | Manzoni says he took the events told in the novel from a manuscript by an anonymous author |
| 6 | Sebbene il tema della peste domini gran parte della narrazione, nessuno tra i personaggi principali decede a causa della malattia | Although the theme of the plague dominates much of the narrative, none of the main characters die of the disease |
| 7 | Gertrude, la Monaca di Monza, scelse di prendere i voti per la sua grande vocazione religiosa | Gertrude, the Nun of Monza, chose to take her vows for her great religious vocation |
| 8 | L’Innominato si pente delle proprie malefatte e si converte in seguito al suo incontro con la Monaca di Monza | The Unnamed repents of his misdeeds and converts following his meeting with the Nun of Monza |
| 9 | Padre Cristoforo scelse di diventare frate per espiare il proprio passato di violenze | Father Cristoforo chose to become a friar to atone for his past of violence |
| 10 | Dal romanzo traspare la fede di Alessandro Manzoni nella Provvidenza divina | The novel reveals Alessandro Manzoni's faith in Divine Providence |

| Ansiolitici | | Anxiolytics (ANS) |
| --- | --- | --- |
| 1 | Le benzodiazepine, al contrario di altri farmaci ansiolitici, non provocano dipendenza o assuefazione | Benzodiazepines, unlike other anti-anxiety drugs, are not addictive or habit-forming |
| 2 | La sigla SSRI è un acronimo che significa Selective Serotonin Reuptake Inhibitors. | The abbreviation SSRI is an acronym which stands for Selective Serotonin Reuptake Inhibitors |
| 3 | Non è necessario interrompere l’assunzione di benzodiazepine in modo graduale, poiché si tratta di farmaci che non comportano scompensi fisiologici. | It is not necessary to discontinue the intake of benzodiazepines gradually, since these are drugs that do not cause physiological derangements. |
| 4 | Le benzodiazepine possono restare in circolo nel sangue per giorni | Benzodiazepines can stay in the bloodstream for days |
| 5 | I farmaci ansiolitici possono essere prescritti dal medico di base | Anti-anxiety medications can be prescribed by your primary care physician |
| 6 | L’assunzione di benzodiazepine è sicura anche in compresenza di altre sostanze sedative, come ad esempio l’alcol | Taking benzodiazepines is safe even in the presence of other sedative substances, such as alcohol |
| 7 | I farmaci ansiolitici sono l’unico rimedio efficace contro il disturbo d’ansia | Anti-anxiety drugs are the only effective remedy for anxiety disorder |
| 8 | I barbiturici sono spesso utilizzati nell’eutanasia animale, umana, e per eseguire condanne a morte tramite iniezione letale. | Barbiturates are often used in animal and human euthanasia, and to carry out death sentences by lethal injection. |
| 9 | Il Prozac è un farmaco consigliato anche per le donne in gravidanza | Prozac is a drug recommended for pregnant women as well |
| 10 | Gli ansiolitici sono utilizzati anche nel trattamento dell’insonnia | Anxiolytics are also used in the treatment of insomnia |

| Evoluzionismo | | Evolution (DAR) |
| --- | --- | --- |
| 1 | Le fessure branchiali e un accenno di coda sono presenti nell’embrione di tutti i vertebrati | The gill slits and a hint of a tail are present in the embryo of all vertebrates |
| 2 | L’homo di Neanderthal è una specie di homo più antica dell’homo erectus | Neanderthal homo is a species of homo older than homo erectus |
| 3 | Facendo accoppiare tra loro individui che casualmente mostrano una caratteristica fisica (ad esempio una forma speciale delle piume di un colombo) un allevatore può ottenere animali con le caratteristiche desiderate | By mating individuals that randomly show a physical characteristic (for example a special shape of a pigeon's feathers) a breeder can obtain animals with the desired characteristics |
| 4 | L’homo sapiens discende dall’uomo di Neanderthal | Homo sapiens descends from Neanderthal man |
| 5 | Il creazionismo è l’interpretazione per cui le specie viventi sono rimaste inalterate dal momento della loro apparizione. | Creationism is the interpretation that living species have remained unchanged since their appearance. |
| 6 | Il primo naturalista a proporre l’idea di una graduale modificazione delle specie fu Lamarck | The first naturalist to propose the idea of a gradual modification of species was Lamarck |
| 7 | Più gli embrioni di due specie diverse si somigliano, più è stretta è la loro vicinanza in termini evoluzionistici | The more similar the embryos of two different species are, the closer their proximity is in evolutionary terms |
| 8 | È grazie al contributo del naturalista Linneo e a il suo studio delle piante di pisello che si è iniziato a capire come le caratteristiche ereditarie si trasmettono dai genitori alla prole. | It is thanks to the contribution of the naturalist Linnaeus and his study of pea plants that we have begun to understand how hereditary characteristics are transmitted from parents to offspring. |
| 9 | Il fenotipo è l’espressione visibile del genotipo, ovvero l’insieme delle caratteristiche visibili che si manifestano nell’individuo | The phenotype is the visible expression of the genotype, i.e. the set of visible characteristics that manifest themselves in the individual |
| 10 | A un animale che nuota molto potrebbero venire le zampe palmate; la sua prole erediterebbe allora le zampe palmate | An animal that swims a lot might develop webbed feet; her offspring would then inherit webbed feet |

| Immunodeficienza felina | | Feline Immunodeficiency (FIV) |
| --- | --- | --- |
| 1 | Il virus che causa l’AIDS felina può trasmettersi anche all’uomo | The virus that causes feline AIDS can also be transmitted to humans |
| 2 | Dopo la diagnosi di immunodeficienza felina, al gatto restano pochi mesi di vita | After the diagnosis of feline immunodeficiency, the cat has a few months left to live |
| 3 | Il virus che causa l’AIDS felina si trasmette con lo scambio di fluidi organici, come il sangue | The virus that causes feline AIDS is transmitted by the exchange of body fluids, such as blood |
| 4 | Il virus che causa l’AIDS felina resiste nell’ambiente: ci si può quindi contagiare entrando a contatto con un ambiente contaminato | The virus that causes feline AIDS resists in the environment: one can therefore become infected by coming into contact with a contaminated environment |
| 5 | Tutti i gatti con immunodeficienza felina mostrano sintomi legati alla malattia | All cats with feline immunodeficiency show symptoms related to the disease |
| 6 | L’AIDS felina è più frequente nei gatti interi rispetto ai gatti castrati | Feline AIDS is more common in intact cats than in neutered cats |
| 7 | Per diagnosticare l’immunodeficienza felina è necessario un esame delle urine | A urine test is needed to diagnose feline immunodeficiency |
| 8 | I gatti con immunodeficienza felina possono contagiare i cani con cui vivono | Cats with feline immunodeficiency can infect the dogs they live with |
| 9 | Una gatta potrebbe risultare sieropositiva per aver sviluppato gli anticorpi, pur senza avere più il virus in circolo | A cat could be FIV positive for having developed the antibodies, even though she no longer has the virus in her circulation |
| 10 | Il virus della FIV si trasmette frequentemente durante le zuffe territoriali | The FIV virus is frequently transmitted during territorial fights |

### Stimuli

Participants were exposed to two of the three following blocks of news articles. If by the time of data collection a post had become outdated, it was replaced with a news post with similar content but more recent. Furthermore, they saw four extra Facebook posts, consisting of pictures of animals, working as distractors. All the posts were presented in a randomized order.

**High self-involvement.**

Climate change (GW)

[https://www.liberoquotidiano.it/news/commenti-e-opinioni/28857250/greta-thunberg-se-stai-con-lei-stai-con-rincari-danni-collaterali-lotta-ambiente.html](about:blank)

[https://www.fanpage.it/esteri/clima-polizia-vieta-le-proteste-di-extinction-rebellion-a-londra-gia-1-400-arresti/](about:blank)

[https://www.ilfattoquotidiano.it/2022/05/20/perquisizioni-ai-giovani-di-fridays-for-future-a-milano-dopo-denuncia-gazprom-fatto-spogliare-e-obbligato-a-fare-anche-piegamenti-durante-blitz-dei-carabinieri/6598678/](about:blank)

[https://palermo.repubblica.it/cronaca/2021/08/11/news/a_siracusa_48_8_gradi_mai_una_temperatura_cosi_alta_in_europa-313709104/](about:blank)

[https://www.repubblica.it/green-and-blue/2021/08/04/news/siberia_serbatorio_di_metano_il_caldo_aumenta_le_fuoriuscite_dal_permafrost-312843405/](about:blank)

[https://www.repubblica.it/green-and-blue/2022/03/03/news/clima_australia_alluvione_inondazioni_eventi_meteo_estremi-340097347/](about:blank)

[https://www.nationalgeographic.it/ambiente/2021/04/26-modi-per-ridurre-il-nostro-impatto-sul-pianeta](about:blank)

Abortion (IVG)

El Salvador, condannata a 30 anni per un aborto spontaneo (nextquotidiano.it)

Giorgia Soleri, fidanzata di Damiano dei Maneskin: «Ho abortito a 21 anni», chi era il padre del bambino? - DonnaPOP

In Italia ci sono almeno 31 strutture con il 100% di medici obiettori - Il Post

https://www.ilmessaggero.it/mondo/amazon_stati_uniti_aborto_rimborso-6668091.html

Il potere delle multinazionali: Amazon pagherà le donne che abortiscono (provitaefamiglia.it)

Diritto all'aborto. L'arcivescovo di San Francisco attacca Nancy Pelosi e le nega la comunione (rainews.it)

"L'aborto non è un contraccettivo ma rimane un diritto delle donne" - La Ragione

https://www.wired.it/attualita/politica/2020/10/01/cimitero-feti-roma/

**Medium Self-Involvement.**

Evolutionism (DAR)

[https://www.corriere.it/tecnologia/22_febbraio_11/darwin-day-2022-12-febbraio-213-anni-fa-nasceva-padre-evoluzionismo-6b6c2408-89d9-11ec-ab70-14f9e3dc0d34.shtml](about:blank)

[https://www.rivieraoggi.it/2011/02/01/112597/sempre-meno-evoluzionismo-nelle-scuole-usa-e-in-italia/](about:blank)

[https://pikaia.eu/ci-risiamo-il-ministro-delleducazione-indiano-rinnega-la-teoria-dellevoluzione/](about:blank)

[https://www.lastampa.it/cronaca/2019/11/07/news/l-anello-mancante-tra-scimmie-e-uomini-e-stato-scoperto-in-germania-1.37872096/](about:blank)

[https://www.nationalgeographic.it/scienza/2021/06/trovati-in-cina-i-fossili-di-un-rinoceronte-gigante](about:blank)

[https://www.lescienze.it/mind/2022/03/15/news/recettori_odore_corpo_muschio_ridotta_sensibilita_olfatto_evoluzione_geni-8958374/](about:blank)

[https://www.lescienze.it/news/2015/07/18/news/evoluzione_confutazione_obiezioni_creazioniste_creazionismo-2694916/](about:blank)

Anxyolitics (ANS)

https://www.federfarma.it/Edicola/Filodiretto/VediNotizia.aspx?id=22353#:~:text=A%20causa%20della%20pandemia%20da,e%20Umbria%20(%2B73%25).

https://www.fanpage.it/attualita/lacquisto-online-di-farmaci-per-ansia-insonnia-e-stress-e-raddoppiato-con-la-pandemia-di-covid/

https://www.fanpage.it/attualita/ansiolitici-nel-cappuccino-alla-collega-rivale-temeva-tagli-del-personale-condannata/

https://www.corriere.it/salute/neuroscienze/14_settembre_29/rischio-alzheimer-aumenta-se-si-prendono-troppi-ansiolitici-0c3f3316-47d4-11e4-85be-0ddddac1a56f.shtml

https://www.aboutpharma.com/scienza-ricerca/benzodiazepine-ad-alte-dosi-labuso-si-diffonde-tra-medici-e-professionisti/

https://www.repubblica.it/salute/2021/12/13/news/prigionieri_degli_ansiolitici_la_dipendenza_da_benzodiazepine-329689036/

https://www.vice.com/it/article/kz5edx/consumo-di-xanax-cosa-sapere

https://psicoadvisor.com/ansiolitici-e-alzheimer-le-benzodiazepine-aumentano-il-rischio-di-demenza-29309.html

https://www.openpolis.it/numeri/il-portogallo-e-primo-in-europa-per-prescrizioni-di-ansiolitici/

**Low Self-Involvement**

I Promessi Sposi (PP)

https://www.corriere.it/scuola/universita/15_novembre_28/promessi-sposi-manzoni-mostra-virtuale-universita-sapeinza-tablet-smartphone-movio-cf3141c4-95c7-11e5-8b73-dd829849c746.shtml

https://www.repubblica.it/serietv/schede/i-promessi-sposi/2651/

https://www.repubblica.it/serietv/2021/06/19/news/una_serie_prequel_dei_promessi_sposi_e_provvidenza_idea_del_produttore_del_trono_di_spade_-306774776/

https://www.eccolecco.it/arte-cultura/musei-gallerie/museo-manzoniano-lecco/

https://www.chiesadimilano.it/news/arte-cultura/la-monaca-di-monza-la-vera-storia-tra-arte-e-letteratura-33678.html

https://www.lavocedeltrentino.it/2020/04/01/coronavirus-e-peste-nei-promessi-sposi-alcune-analogie-che-fanno-riflettere/

https://www.raicultura.it/letteratura/articoli/2018/12/Affinati-e-lInnominato-de-I-Promessi-Sposi-51f560c0-900d-4360-b8aa-b06f959a3b7c.html

https://www.raicultura.it/letteratura/articoli/2018/12/I-promessi-sposi-in-tv-lassalto-ai-forni-a722db9d-9e5d-4414-a52b-b0ccb02369d4.html

https://www.leccotoday.it/notizie/costumi-promessi-sposi.html

Feline Immunodeficiency (FIV)

Accudire un gatto con la FIV, tutti i consigli (ilmiogattoeleggenda.it)

Il parassita della Toxoplasmosi ci rende più belli? | Wired Italia

[https://www.corriere.it/animali/18_luglio_18/io-milo-casetta-gatti-4fe2c03c-8a11-11e8-8bbc-b107b233a106.shtml](about:blank)

[https://www.corriere.it/tecnologia/22_febbraio_18/morta-pot-roast-gatta-piu-amata-tiktok-8fd28646-90bb-11ec-9e8a-badec6e7adb8.shtml](about:blank)

[https://ricerca.repubblica.it/repubblica/archivio/repubblica/2017/05/20/per-i-gatti-antirabbica-e-fiv-da-sapereBologna21.html](about:blank)

[https://www.veggieanimals.com/it/blog/post/DIETA-VEGANA-NELLA-FIV-E-NELLA-FELV.html](about:blank)

[https://www.amoreaquattrozampe.it/gatti/convivenza-gatto-fiv-gatto-sano-strategie/118268/](about:blank)

[https://www.kodami.it/addio-a-pot-roast-famosissima-gatta-su-tiktok-ha-perso-la-battaglia-contro-laids-felina/](about:blank)

[https://www.ilpescara.it/attualita/gatto-micio-gino-peritonite-infettiva-felina-raccolta-fondi.html](about:blank)

### Questionnaires and assessments

Participants were asked to fill up the following questionnaires, administrated in a faithful Italian translation.

Each item of the selected scales was framed as follows: “How much do you agree with the following statements?”, and participants were asked to answer using a 0 – 100 VAS going from 0 = *Totally disagree* to 100 = *Totally agree*.

**Cognitive style** (Cacioppo & Petty, 1982; Epstein et al., 1996)

| I don't like to have to do a lot of thinking. | Non mi piace dover pensare molto |
| --- | --- |
| I try to avoid situations that require thinking in depth about something. | Cerco di evitare situazioni che richiedono riflessioni approfondite |
| I prefer to do something that challenges my thinking abilities rather than something that requires little thought. | Preferisco fare qualcosa che sfida le mie capacità di ragionamento piuttosto che qualcosa che richiede poca riflessione |
| I prefer complex to simple problems. | Preferisco i problemi complessi a quelli semplici |
| Thinking hard and for a long time about something gives me little satisfaction. | Pensare intensamente e a lungo a qualcosa mi dà poca soddisfazione |
| I trust my initial feelings about people. | Mi fido delle mie impressioni iniziali sulle persone |
| I believe in trusting my hunches. | Credo molto nel mio intuito. |
| My initial impressions of people are almost always right. | Le mie prime impressioni sulle persone sono quasi sempre giuste |
| When it comes to trusting people, I can usually rely on my "gut feelings." | Se devo fidarmi delle persone, di solito posso affidarmi alle mie sensazioni di pancia. |
| I can usually feel when a person is right or wrong even if I can't explain how I know | Di solito riesco a intuire quando una persona ha ragione o torto, anche se non riesco a spiegare come lo so |

**Political view** (Kahan, 2012)

| Il governo interferisce troppo nella nostra vita quotidiana. | The government interferes far too much in our everyday lives. |
| --- | --- |
| A volte il governo ha bisogno di fare leggi che impediscano alle persone di farsi del male. | Sometimes government needs to make laws that keep people from hurting themselves. |
| Il governo dovrebbe smettere di dire alla gente come vivere la propria vita. | It's not the government's business to try to protect people from themselves. |
| Il governo dovrebbe fare di più per portare avanti gli obiettivi della società, anche se questo significa limitare la libertà e le scelte degli individui. | The government should do more to advance society's goals, even if that means limiting the freedom and choices of individuals. |
| Per il bene della società, il governo dovrebbe porre dei limiti alle scelte che gli individui possono compiere. | Government should put limits on the choices individuals can make so they don't get in the way of what's good for society. |
| Siamo andati troppo oltre nel promuovere uguali diritti in questo Paese. | We have gone too far in pushing equal rights in this country. |
| La nostra società sarebbe migliore se la distribuzione della ricchezza fosse più equa. | Our society would be better off if the distribution of wealth was more equal. |
| Dobbiamo ridurre drasticamente le disuguaglianze tra ricchi e poveri, bianchi e persone di colore, uomini e donne. | We need to dramatically reduce inequalities between the rich and the poor, whites and people of color, and men and women. |
| La discriminazione contro le minoranze è ancora un problema molto serio nella nostra società. | Discrimination against minorities is still a very serious problem in our society. |
| Sembra che le persone di colore, le donne, gli omosessuali e altri gruppi non vogliano diritti uguali, ma vogliano diritti speciali solo per loro. | It seems like blacks, women, homosexuals and other groups don't want equal rights, they want special rights just for them. |
| La società nel suo complesso è diventata troppo morbida e accomodante. | Society as a whole has become too soft and feminine. |

**Social media use** (Orosz et al., 2016)

| If I could visit only one site on the Internet, it would be Facebook. | Se potessi visitare un solo sito su internet, quel sito sarebbe Facebook. |
| --- | --- |
| I feel bad if I don't check my Facebook daily. | Mi sento a disagio se non controllo il mio Facebook ogni giorno. |
| Before going to sleep, I check Facebook once more. | Controllo Facebook prima di andare a dormire. |
| I spent time on Facebook at the expense of my obligations. | Passo il mio tempo su Facebook al posto di fare quello che devo. |
| I spend more time on Facebook than I would like to. | Passo su Facebook più tempo di quanto vorrei. |
| It happens that I use Facebook instead of sleeping. | Mi capita di stare su Facebook invece di dormire. |

**Intellectual humility** (Leary et al., 2017)

| I question my own opinions, positions, and viewpoints because they could be wrong | Metto in dubbio le mie opinioni, posizioni e punti di vista perché potrebbero essere sbagliati |
| --- | --- |
| I reconsider my opinions when presented with new evidence | Riconsidero le mie opinioni quando mi vengono presentate nuove prove |
| I recognize the value in opinions that are different from my own | Riconosco il valore delle opinioni diverse dalle mie |
| I accept that my beliefs and attitudes may be wrong | Accetto che le mie convinzioni e i miei atteggiamenti possano essere sbagliati |
| In the face of conflicting evidence, I am open to changing my opinions | Di fronte a prove contrastanti, sono disposto a cambiare le mie opinioni |
| I like finding out new information that differs from what I already think is true. | Mi piace scoprire nuove informazioni che differiscono da ciò che già penso sia vero. |

**Attitudes**

| Penso sia fondamentale controllare i propri gatti per ogni possibile malattia felina | I think it is essential to check your cats for any possible feline diseases |
| --- | --- |
| L'immunodeficienza felina è meno grave di quello che sembra | Feline immunodeficiency is less serious than it seems |
| Penso che I Promessi Sposi siano una delle opere più importanti della storia della letteratura italiana | I think that The Betrothed is one of the most important works in the history of Italian literature |
| Penso che lo studio (insegnamento) de I Promessi Sposi andrebbe sostituito con lo studio di altre opere più interessanti | I think that the study (teaching) of The Betrothed should be replaced with the study of other more interesting works |
| A scuola c'è troppa insistenza sulle teorie evoluzionistiche | At school there is too much insistence on evolutionary theories |
| Penso che la conoscenza dell’evoluzionismo andrebbe diffusa di più | I think that knowledge of evolutionism should be spread more |
| Penso che gli ansiolitici siano da usare con molta attenzione | I think anxiolytics should be used very carefully |
| Penso che gli ansiolitici rendano molto più facile la vita delle persone | I think anti-anxiety medications make people's lives a lot easier |
| Penso che si debba compiere ogni sforzo possibile per contrastare i cambiamenti climatici | I think every possible effort should be made to combat climate change |
| Penso che la situazione climatica sia meno grave di quel che si dice | I think the climate situation is less serious than people say |
| L’aborto è una pratica eticamente sbagliata | Abortion is an ethically wrong practice |
| Penso sia necessario facilitare l’accesso all’aborto | I think it is necessary to facilitate access to abortion |

**Self-involvement**

- Quanto ti senti coinvolto/a dall’argomento? (How much do you feel involved by the topic?)
  Per nulla coinvolto/a – Estremamente coinvolto/a
- Quanto ti impegneresti in una discussione online sull’argomento? (How much would you be willing to discuss the topic in an online conversation?)

Per nulla - Totalmente

**Perceived knowledge**

- Quanto pensi di saperne sull’argomento? (How much do you think you know about the topic?)
  Nulla – Tutto

### References

Bartholomew Eldredge, L. K., Markham, C. M., Ruiter, R. A. C., Fernández, M. E., Kok, G., & Parcel, G. S. (2016). *Planning health promotion programs, an Intervention Mapping approach* (4th ed.). Jossey-Bass Inc.

Cacioppo, J. T., & Petty, R. E. (1982). The need for cognition. *Journal of Personality and Social Psychology*, *42*(1), 116–131. https://doi.org/10.1037/0022-3514.42.1.116

Epstein, S., Pacini, R., Denes-Raj, V., & Heier, H. (1996). Tversky & Kahneman, 1983), schematic (Leventhal, 1984), prototypical (Rosch, 1983), narrative (Bruner, 1986), implicit (Weinberger & McClelland, 1991), imagistic-nonverbal (Bucci, 1985; Paivio, 1986), experiential (Epstein, 1983), mythos (Labouvie-Vief, 1990), and first-sig-nal system (Pavlov, cited in Luria, 1961) and the other as think-ing-conceptual-logical (Buck. In *Journal of Personality and Social Psychology* (Vol. 71, Issue 2). Labouvie-Vief.

Kahan, D. M. (2012). Cultural Cognition as a Conception of the Cultural Theory of Risk. In *Handbook of Risk Theory* (pp. 725–759). Springer Netherlands. https://doi.org/10.1007/978-94-007-1433-5_28

Leary, M. R., Diebels, K. J., Davisson, E. K., Jongman-Sereno, K. P., Isherwood, J. C., Raimi, K. T., Deffler, S. A., & Hoyle, R. H. (2017). Cognitive and Interpersonal Features of Intellectual Humility. *Personality and Social Psychology Bulletin*, *43*(6), 793–813. https://doi.org/10.1177/0146167217697695

Lord, C. G., Ross, L., & Lepper, M. R. (1979). Biased assimilation and attitude polarization: The effects of prior theories on subsequently considered evidence. *Journal of Personality and Social Psychology*, *37*(11), 2098–2109. https://doi.org/10.1037/0022-3514.37.11.2098

Orosz, G., Tóth-Király, I., & Bőthe, B. (2016). Four facets of Facebook intensity — The development of the Multidimensional Facebook Intensity Scale. *Personality and Individual Differences*, *100*, 95–104. https://doi.org/10.1016/j.paid.2015.11.038

Toplak, M. E., West, R. F., & Stanovich, K. E. (2014). Assessing miserly information processing: An expansion of the Cognitive Reflection Test. *Thinking and Reasoning*, *20*(2), 147–168. https://doi.org/10.1080/13546783.2013.844729

1. In our preregistration, we defined this variable “attitude strength”. However, this definition is in contrast with conventional definitions in social psychology, where attitude strength is measured as the certainty and confidence in one’s opinion, independently of the extremity or moderation of such opinion (Fabrigar et al., 2005). Therefore we decided to refer to our measure not as “attitude strength”, but, more accurately, as the “opinion extremity”. [↑](#footnote-ref-1)
2. The term “topics of different levels of self-involvement” could be interpreted ambiguously—as either different content topics or as the involvement-based categorization (high, medium, low). To clarify: the regression model included the three involvement groups as levels of the predictor, not the individual topics themselves. We have added a clarification in Table 1 accordingly. While this clarification does not alter the analysis or results, we include it here for transparency. [↑](#footnote-ref-2)
